# Tubercular Disease of the Abdomen

**Published:** 1831

**Authors:** 


TUBERCULAR DISEASE OF THE ABDOMEN. By Mr. SWIFT.

[Richmond Hospital.]

THE following case is very clearly and ably reported by Mr. Swift, in a late Number of our contemporary, the Medical and Surgical Journal.

*Case.* "Thomas Malone, aged 36 years, was admitted into the Richmond Hospital on the 24th of September, 1829, with considerable swelling of the abdomen, and decided marks of the existence of fluid within its cavity. On making pressure, a large flattened tumour, of firm consistence and irregular surface, was discovered occupying the epigastric, hypochondriac, and part of the umbilical regions, and terminating below by a well-defined margin, unequally convex, and notched, so as to bear a close resemblance to the anterior edge of the liver. The correspondence however, was not exact, the tumour could be nearly insulated superiorly, and it was more moveable than an enlarged liver would have been. Tumours equally firm, but more irregular and knotty, could be felt in the iliac fossæ, and in the lower part of the umbilical and hypogastric regions the central one particularly was moveable. There was scarcely any pain on making strong pressure, or after the closest examination, nor had the origin or progress of the disease been attended with any remarkable suffering. He complained of a sense of great weight at the stomach after taking any quantity of food, particularly animal, and stated, that vegetables produced the most distressing flatulence. If he ate heartily, the stomach generally rejected its contents in about an hour after his meal; but if the portion of aliment used was sparing and light, or if vomiting had not taken place about the period mentioned, the process of digestion was completed. His pulse was soft, rather feeble, and varied from 80 to 90; he was affected with slight dyspnœa, which was materially increased by stooping; his tongue white, bowels costive, and subject to borborygmi. The dryness and harshness of his skin was remarkable; his urine was scanty (about a pint in twenty-four hours), and his thirst considerable. He slept badly, and, though not confined to bed, was languid and incapable of exertion. His form was much wasted, and his countenance pale, yellow, and stamped with that peculiar expression which long-continued suffering, however trivial, constantly impresses. There was no affection of the cerebro-spinal system.

About two years from the date of his admission (previous to which he enjoyed excellent health), he became infected with syphilis, from wearing a sailor's trowsers, purchased at a cast-clothes shop. The disease first appeared on the scrotum, next on the penis, and, in some time afterwards, he was attacked by heaviness and severe pains, chiefly in the shoulders, succeeded by an eruption of the papular kind, on his legs, arms, and forehead. For these symptoms, which he was informed were venereal, he took mercury for three weeks, was salivated, and got apparently well. While under the influence of mercury, he drank very freely, and was exposed to intense heats, being employed about the stove of a rectifying distillery.— From this time up to what he considers the commencement of his present disease, he became affected with chills, flushes of heat, perspirations, debility and loss of appetite. Four or five months afterwards, during the active prevalence of these symptoms, swelling of the abdomen took place, with obstinate costiveness, piles, scanty urine, sensation of weight in the epigastrium after taking any considerable quantity of food, and pains shooting from the cartilage of the ninth rib, towards the lumbar vertebræ, on the left side. About three months afterwards his breathing became difficult, he experienced pain in the left side of the epigastrium on stooping, and then, for the first time, felt the tumour in the abdomen. He stated, that, when he discovered it, he thought it might be two palms breadth in extent, and that it had been progressively increasing since. For the reasons which have been mentioned before, he has avoided animal and vegetable diet; and for the last six months, lived almost exclusively on tea and slops. Four or five days generally intervened between each alvine evacuation; but the colour of the stools is natural. He frequently hears a loud rumbling noise in the great intestine, accompanied with pain round the lower part of the abdomen, which he regards as a call to stool, but is never affected with tenesmus. Flatulence is a source of great annoyance to him, and he feels more relieved by the expulsion of wind than fæcal evacuation. About three months before admission, he had bilious vomiting, which continued for three weeks, attacking him every second or third morning. He went into Stevens's Hospital some time afterwards, and got hydragogue purgatives, and a solution of cream of tartar, which drink he thinks reduced the size of the abdomen, but gave no relief to the other symptoms.

From the date of his admission to the 6th of October, his treatment consisted in friction with volatile liniment to the abdomen, purgative draughts, blue pill occasionally, and enemata, which gave some slight relief.

Oct. 7th. To have the belly rubbed with the same liniment, combined with tartarized antimony and ung. hydrarg.

13th. Potio potassæ super. tartrat. ad libitum. Two of the following pills to be taken night and morning:—

℞ Pil. rhei, ℈ij. Ext. colocynth. ij. Capsici, gr. v. Olei carui, ♏v. Ft. pil. x.

22d. Has two evacuations daily since he has been using the pills. Occasional tenesmus; flatulence less troublesome; thirst diminished. Urinary secretion as before; tongue and appetite unimproved. The liniment has produced an extensive crop of pustules over the abdomen.

31st. Complains of pain in the left side of the epigastrium; to rub the liniment as before over the affected part.

4th Nov. Taking his pills, which produce two motions as before, the last scanty, and attended with griping; abdomen tense; tumour undiminished.

6th. Pain in the left hypochondrium, again complains of want of sleep. Countenance pale and sunken. Diarrhœa with tenesmus; the stools thin, slimy, and greenish; some irritability of bladder. Tumour as before; no œdema of the lower extremities. The diarrhœa continued for about a week, and the stools were occasionally tinged with blood. It ceased on the 14th.

24th. Dyspnœa increased. It affects him now when he lies on his left side; appetite worse—emaciation increasing. Abdomen tympanitic.

16th Dec. Frequent vomiting almost immediately after taking food; œdema of the left lower extremity.

29th. Dyspnœa increasing. Numbness of the left thigh, with progressive œdema and coldness of the entire limb; more emaciation; vomiting as on the 16th, which continued until the 5th of January, when it was checked.

Jan. 9th, 1830. Has been using friction with volatile liniment over the left lower extremity. Ordered ℥j. of the following mixture:—4tis horis

Mist. camphoræ, ℥iv. Spirit. ammon. aromat. ʒj. Sp. etheris oleosi, ℥iij. M.

14th. To have his mixture repeated; œdema increasing.

23d. Swelling of the left lower extremity very great; œdema commencing in the right leg and foot. Vesications have formed on the anterior part of the left leg, and burst, discharging a large quantity of serous fluid and emptying the limb. The integuments surrounding the vesicles are of purplish colour. Fluid in the abdomen much lessened, and the surface and figure of the diseased mass rendered more evident. No sleep; increasing debility. To have opii, gr. jss. o. n. ℔ss. of decoct. genistæ, with half an ounce of supertartrate of potass daily; his draughts as before occasionally; a poultice p. d.

26th. Cuticle extensively separated from the anterior part of the left leg.

From the toes to the upper part of the ulcer, the surface around presents an erysipelatous blush. The sore itself is of a deeper red, and is coated over with a layer of lymphy matter.

29th. Sloughing of the limb rapidly increasing—dyspnœa aggravated. Has been lying on his right side for the last few days. Great thirst; insomnolence and exhaustion; pulse cannot be felt—is evidently sinking.

30th. Died.

*Necroscopic Phenomena.*—On examination, the thorax and upper extremities appeared much emaciated; the abdomen swollen, with distinct sense of fluctuation, and the lower extremities affected with extensive anasarca. On cutting into the abdomen an immense quantity of fluid gushed out, of a serous character and reddish tinge, and closely resembling that which is effused in ascites. In this fluid was seen floating a vast number of colourless or whitish transparent bodies, of a flattened, spheroidal, or lenticular form, and of the average size of a small hazel-nut, in each of which were observed spots of minute vascularity. There was some variety in the degree of transparency, and on holding them to the light, a slightly opake spot was observed in the centre of each. The consistence and firmness possessed by these tubercles was that of the healthy lens in the young subject. An immense tumour occupying the situation, and possessing the dimensions of the omentum, now presented itself to view, extending over the epigastric, hypochondriac, and part of the umbilical and lumbar regions. To the surface of this tumour, a number of the bodies before mentioned adhered slightly, and could be detached from it by friction. It was covered by a membrane resembling those of the serous class, but it was impossible to trace satisfactorily its exact continuity and identity with the sound peritoneum. A delicate membrane, of similar character, invested each of the small bodies.

A section of the tumour from behind forwards shewed it to vary from an inch to two inches in thickness, and to consist of a number of cells, each capable of containing about the bulk of a garden-pea, and filled with a transparent gelatinous fluid. From the analogy of their appearance, size, and contents, to those of the small bodies described before, it appeared evident that the tumour was formed by the successive concretion of a vast number of tubercles. Masses of the same kind, of smaller size, were found occupying the situation of the mesentery, and the duplicatures which attach the different portions of the colon and its sigmoid flexure. There were no particular marks of disease in the liver, except that it was very much reduced in size and condensed in structure, from the pressure of the tumour, by which it had been forced up considerably towards the right side of the chest. The spleen was quite healthy. The stomach and intestinal canal presented no appearance of derangement worthy of remark. A few tubercles were found in the cavity of the thorax."

A chemical examination of the tumours was made by Mr. Upjohn, and it was concluded that they consisted chiefly of gelatine.

